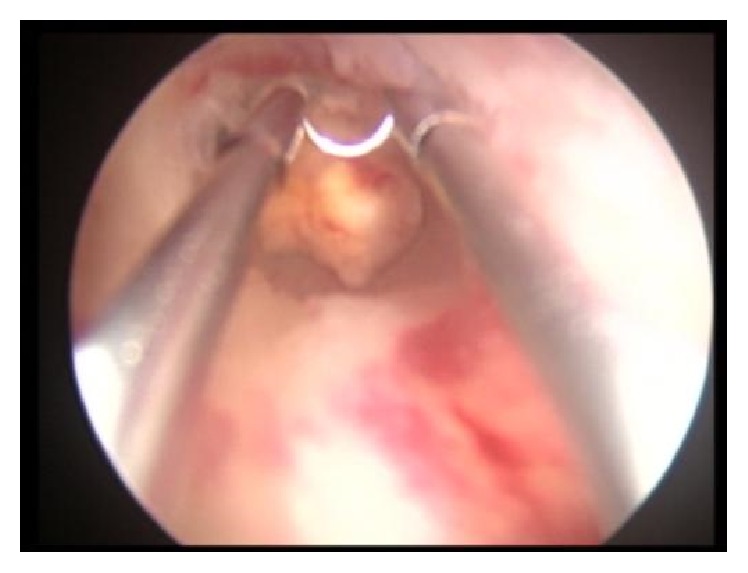# Conservative Resectoscopic Surgery, Successful Delivery, and 60 Months of Follow-Up in a Patient with Endometrial Stromal Tumor with Sex-Cord-Like Differentiation

**DOI:** 10.1155/2016/5736865

**Published:** 2016-04-26

**Authors:** Pasquale De Franciscis, Flavio Grauso, Domenico Ambrosio, Marco Torella, Enrico Michelino Messalli, Nicola Colacurci

**Affiliations:** Department of Women, Child and General and Specialized Surgery, Second University of Naples, Largo Madonna delle Grazie 1, 80138 Napoli, Italy

## Abstract

Uterine tumors with sex-cord-like differentiation are extremely rare types of uterine stromal neoplasm. These tumors were classified into two groups with considerable practical relevance because clinical behaviour of uterine tumor resembling ovarian sex cord tumor (UTROSCT) differs widely from its closely related endometrial stromal tumors with sex-cord-like elements (ESTSCLE). Treatment and prognosis of these tumors are unresolved issues because of the exiguous number of reported cases. We describe a rare case of endometrial stromal tumor with sex-cord-like differentiation successfully treated by resectoscopic surgery and conservation of the uterus, in an infertile patient affected by metrorrhagia. This procedure resulted in a pregnancy immediately after treatment and in a successful delivery. During 60 months of follow-up no evidence of recurrence was observed.

## 1. Introduction

Uterine tumors with sex-cord-like differentiation are extremely rare types of uterine stromal neoplasm. Treatment and prognosis of these tumors are unresolved issues because of the exiguous number of reported cases. We describe a rare case of endometrial stromal tumor with sex-cord-like differentiation successfully treated by resectoscopic surgery and conservation of the uterus, in an infertile patient affected by metrorrhagia.

## 2. Case Presentation

**Figure 1:**
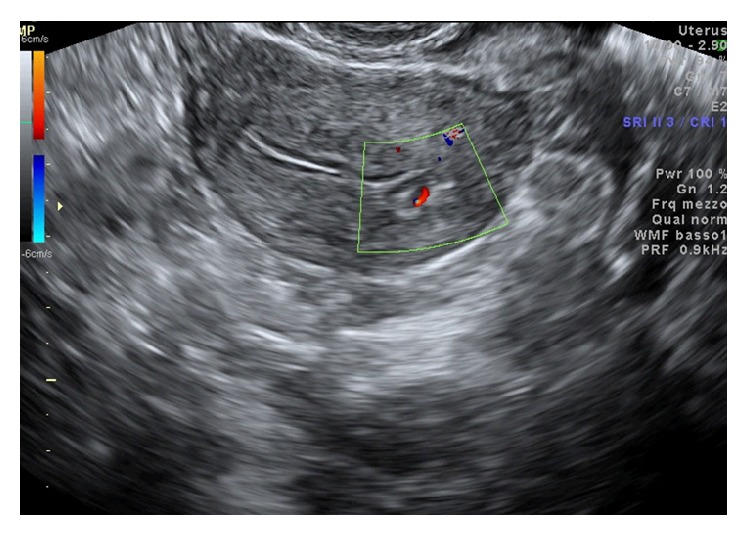


A 38-year-old nulligravida woman presented with metrorrhagia 5 months ago and primary infertility at Outpatient Fertility Clinic of the Second University of Naples. The sonohysterography (Figure 1) showed a 8 × 7 mm-sized echoic nodular mass protruding in the cavity to 60–70%, without abnormal vascularisation. The endometrial implant of the lesion was 5 mm in diameter without infiltration of myometrial layer. The patient underwent diagnostic hysteroscopy that showed a 10 mm-sized yellowish grey lesion localized in the endometrial cavity in proximity of left tubal orifice. A biopsy was performed reaching the pathological diagnosis of endometrial stromal tumor with sex-cord-like differentiation. According to the immunohistochemical results the neoplastic cells were positive for calretinin, desmin, and smooth muscle actin and focally positive for melan A, WT1, inhibin, and Pan-Cytokeratin. The different treatment options were discussed with the patient. A conservative resectoscopic surgery was chosen to preserve fertility, despite the risk of incomplete removal of neoplastic tissue. Surgery was scheduled in the proliferative phase of the next menstrual cycle, reaching apparent complete excision of the lesion (Figure 2). Histological examination and immunohistochemistry confirmed the initial diagnosis of endometrial stromal tumor with sex-cord-like differentiation; such lesion was not classifiable in any subgroup because of fragmentation and small size of the sample. The diagnosis was confirmed without additional elements by two other National Pathological Services. Two months after surgery, a serum pregnancy test resulted positive and the pregnancy continued to progress without complications. At 39 weeks and 3 days an elective caesarean section on maternal request was performed and a healthy baby was delivered. The option for hysterectomy was discussed but the patient refused it. Therefore, a careful follow-up was scheduled performing a transvaginal ultrasound examination every six months and a diagnostic hysteroscopy every year. After 60 months of follow-up, no recurrence was observed.

## 3. Discussion

Uterine tumors with sex-cord-like differentiation are extremely rare type of uterine tumor and its clinical features are not fully understood. The classification, proposed by Clement and Scully [1] in 1976, has considerable practical relevance because clinical behaviour of uterine tumor resembling ovarian sex cord tumor (UTROSCT) differs widely from its closely related endometrial stromal tumor with sex-cord-like elements (ESTSCLE). The latter behave similar to endometrial stromal sarcoma with a propensity for wide local and distant metastasis. On the other hand UTROSCT is a benign tumor with occasional local recurrence [2]. However, the preoperative diagnosis of UTROSCT is very difficult because there are no specific imaging findings. This type of tumor is usually diagnosed based on the histomorphologic features including a predominant pattern of the cords, nests, and trabeculae resembling granulosa or Sertoli cell tumors of the ovary. The diagnosis is confirmed with immunohistochemical staining characterized by the coexpression of epithelial, smooth muscle, sex cord markers, and steroid receptors [3–6]. UTROSCT has long-term clinical behaviour less aggressive than ESTCLE and should be considered as neoplasm with an uncertain, but probably low, malignant potential [3, 4, 7–9]. Such classification of UTROSCT into two histologic subtypes is relatively new, and a large portion of literature refers to UTROSCT generally without subcategorization [8]. Moreover the World Health Organization classification defines UTROSCT as miscellaneous tumor and ESTSCLE is considered as rare variant of endometrial stromal tumor. Establishing a generally agreed management is complicated by the rarity of cases and ambiguous classification. Total hysterectomy with bilateral adnexectomy is the most common treatment, followed by total hysterectomy alone and mass resection alone [8], in accordance to age and parity. Conservative management should be considered with caution in young women who have a strong desire for fertility preservation. The present is the fourth case of the literature describing a pregnancy after a resectoscopic surgery for an endometrial stromal tumor with sex-cord-like differentiation; notably it is the first report of a conservative management carried out despite the impossibility to classify the tumor in type I or type II, with a subsequent pregnancy obtained spontaneously and furthermore a childbirth not followed by a total hysterectomy. Therefore a careful follow-up was scheduled and still carried out after 60 months. In the literature no recurrence is reported among patients who were conservatively managed during a variable follow-up (13 months to 7 years) [7, 10–14]. According to Blake et al., the risk factors for disease free survival in UTROSCT are related to pelvic pain at the time of diagnosis, Clement type I tumor (ESTSCLE), tumor size >10 cm, presence of cervical/extrauterine disease, and lymphovascular space invasion [8]. According to Pradhan and Mohanty [9], infiltrative border, vascular invasion, frequent mitotic figures, serosal rupture, stromal predominance, and cytologic atypia were associated with recurrence of UTROSCT. In conclusion, more research is needed to better characterize endometrial stromal tumor with sex-cord-like differentiation. To date, little available data in the literature cannot lead to a generally agreed management, but a conservative management should be considered in selected cases according to age, parity, and associated risk factors. The safety of this conservative approach needs to be evaluated through a longer follow-up.

## Figures and Tables

**Figure 2:**